# Supplementary material for: Insight into the Causal Relationship between Gut Microbiota and Back Pain: A Two Sample Bidirectional Mendelian Randomization Study
Source: Adv Genet (Hoboken). 2023 Nov 8;4(4):2300192. doi: 10.1002/ggn2.202300192 (PMC10716053; doi:10.1002/ggn2.202300192)
Supplement: Supplementary file 1 — Supporting Information [file GGN2-4-2300192-s001.pdf]

ADVANCED GENETICS
Open Access

Supporting Information

for *Advanced Genetics*, DOI 10.1002/ggn2.202300192

Insight into the Causal Relationship between Gut Microbiota and Back Pain: A Two Sample Bidirectional Mendelian Randomization Study

Jingni Hui, Yujing Chen, Chun'e Li, Yifan Gou, Ye Liu, Ruixue Zhou, Meijuan Kang, Chen Liu, Bingyi Wang, Panxing Shi, Shiqiang Cheng, Xuena Yang, Chuyu Pan, Yumeng Jia, Bolun Cheng, Huan Liu, Yan Wen and Feng Zhang**

Supplement Table 1 .the results of validation Mendelian randomization

Exposure	Outcome	Method	Nsnp	*P*	OR(95%CI)
	Back pain	MR Egger	7	0.954	0.987(0.651,1.498)
class.Gammaproteobacteria.id.3303	Back pain	Weighted median	7	0.329	0.922(0.782,1.086)
	Back pain	IVW	7	0.012	0.861(0.745,0.978)
	Back pain	MR Egger	10	0.019	1.152(1.048,1.266)
class.Melainabacteria.id.1589	Back pain	Weighted median	10	0.078	1.044(0.995,1.094)
	Back pain	IVW	10	0.011	1.047(1.012,1.082)
	Back pain	MR Egger	5	0.526	0.951(0.829,1.091)
family.Actinomycetaceae.id.421	Back pain	Weighted median	5	0.088	0.943(0.881,1.009)
	Back pain	IVW	5	0.004	0.939(0.896,0.982)
	Back pain	MR Egger	17	0.574	1.113(0.772,1.606)
family.Coriobacteriaceae.id.811	Back pain	Weighted median	17	0.042	1.137(1.005,1.286)
	Back pain	Inverse variance weighted	17	0.005	1.111(1.038,1.184)
	Back pain	MR Egger	9	0.059	1.119(1.015,1.234)
family.unknownfamily.id.1000001214	Back pain	Weighted median	9	0.032	1.053(1.004,1.104)
	Back pain	IVW	9	<0.001	1.063(1.036,1.090)
	Back pain	MR Egger	19	0.452	0.966(0.884,1.055)
family.Veillonellaceae.id.2172	Back pain	Weighted median	19	0.422	0.979(0.929,1.031)
	Back pain	IVW	19	0.045	0.961(0.922,1.000)
	Back pain	MR Egger	16	0.434	1.036(0.951,1.128)
genus.Eubacteriumhalliigroup.id.11338	Back pain	Weighted median	16	0.063	1.054(0.997,1.115)
	Back pain	IVW	16	0.005	1.051(1.016,1.085)
	Back pain	MR Egger	7	0.398	1.261(0.771,2.061)
genus.Eubacteriumeligensgroup.id.14372	Back pain	Weighted median	7	0.163	1.114(0.957,1.296)
	Back pain	IVW	7	<0.001	1.130(1.085,1.176)
	Back pain	MR Egger	14	0.032	0.847(0.742,0.968)
genus.Intestinibacter.id.11345	Back pain	Weighted median	14	0.198	0.962(0.907,1.020)
	Back pain	IVW	14	0.018	0.953(0.913,0.993)
	Back pain	MR Egger	6	0.645	1.137(0.686,1.884)
genus.Lachnospira.id.2004	Back pain	Weighted median	6	0.026	0.895(0.812,0.987)
	Back pain	IVW	6	0.050	0.922(0.840,1.003)
	Back pain	MR Egger	8	0.229	1.234(0.907,1.678)
genus.FamilyXIIIUCG001.id.11294	Back pain	Weighted median	8	0.131	1.116(0.968,1.287)
	Back pain	IVW	8	0.019	1.137(1.030,1.243)
	Back pain	MR Egger	13	0.880	1.016(0.831,1.242)
genus.LachnospiraceaeFCS020group.id.11314	Back pain	Weighted median	13	0.108	1.089(0.981,1.208)
	Back pain	IVW	13	0.004	1.085(1.029,1.141)
	Back pain	MR Egger	17	0.574	1.113(0.772,1.606)
order.Coriobacteriales.id.810	Back pain	Weighted median	17	0.042	1.137(1.005,1.286)
	Back pain	IVW	17	0.005	1.111(1.038,1.184)
	Back pain	MR Egger	8	0.840	0.985(0.855,1.134)
phylum.Cyanobacteria.id.1500	Back pain	Weighted median	8	0.146	1.043(0.986,1.103)
	Back pain	IVW	8	<0.001	1.066(1.030,1.101)

Abbreviations:

Nsnp Number of SNPs involved in the analysis

P p value of effect estimate

OR odds ratio

CI confidence interval

IVW Inverse variance weighted
